# Chronic Lymphocytic Inflammation with Pontine Perivascular Enhancement Responsive to Steroids, with Cranial and Caudal Extension

**DOI:** 10.1155/2017/2593096

**Published:** 2017-05-14

**Authors:** Mahmood Mubasher, Aseel Sukik, Ahmed Hassan El Beltagi, Ali Rahil

**Affiliations:** Hamad Medical Corporation, Doha, Qatar

## Abstract

A 23-year-old lady presented with vertigo and imbalance in walking, blurring of vision, diplopia, and headache, in addition to numbness in the lower limbs over a period of six days. On examination patient had nystagmus, ataxia, positive Romberg test, and hyperreflexia. MRI examination of the brain and spinal cord showed evidence of faint bright signal intensity foci in T2/FLAIR involving bilateral cerebral hemispheres, subcortical deep white matter, bilateral thalami, posterior pons and left brachium pontis, and basal ganglia, with small nodular enhancement that aligned along curvilinear structures; those lesions also were apparent along the spinal cord at multiple levels. The clinical and radiological features suggested CLIPPERS (chronic lymphocytic inflammation with pontine perivascular enhancement responsive to steroids) syndrome. Symptoms improved dramatically with high dose oral corticosteroids. Our report addresses the radiological and clinical pattern of a case of CLIPPERS rhombencephalitis, with added superior and inferior extension to involve the brain and spinal cord, which is to emphasize the importance of raising the awareness of this disease and the combined role of radiologist and physicians for the diagnosis of this potentially treatable entity, responsive to glucocorticosteroid immunosuppression.

## 1. Introduction

Chronic lymphocytic inflammation with pontine perivascular enhancement responsive to steroids (CLIPPERS) is a newly rising inflammatory disease involving the central nervous system (CNS), prominently the brainstem and particularly the pons. It was found that two main required features are needed to label the case as CLIPPERS; one of them is the unique radiological appearance on MRI characterized by punctate and curvilinear gadolinium enhancement “peppering” the pons manifested clinically according to the area that is affected. Another required feature for the diagnosis is the clinical and radiological responsiveness to glucocorticosteroid- (GCS-) based immunosuppression [1].

Since the initial report in 2010 by Pittock et al. [2], tens of new cases have been published [1]. We report a further patient with typical features consistent with this syndrome.

## 2. Case Report

23-year-old Filipino lady, previously healthy, presented to the emergency department with vertigo and imbalance in walking of six days prior to admission; her symptoms were also associated with blurring of vision, diplopia, and headache, in addition to numbness in the lower limbs initially that resolved at the time of presentation. The patient denied history of fever, sweating, or weight loss and history of orogenital ulcers or arthralgia. There was no personal or family history of thrombotic events and she was nonalcoholic. On examination, patient was conscious, oriented, and alert, with apparent right sided and upward jerky nystagmus, but normal range of motion of both eyes, normal pupillary reflexes, and fundoscopy; patient was found to have loss of coordination in the four limbs manifested by abnormal finger nose test and dysdiadokinesia, with abnormal heal shin test, that was more marked on the left side; otherwise motor, tone, touch, and position sensation were all intact.

**Figure 1:**
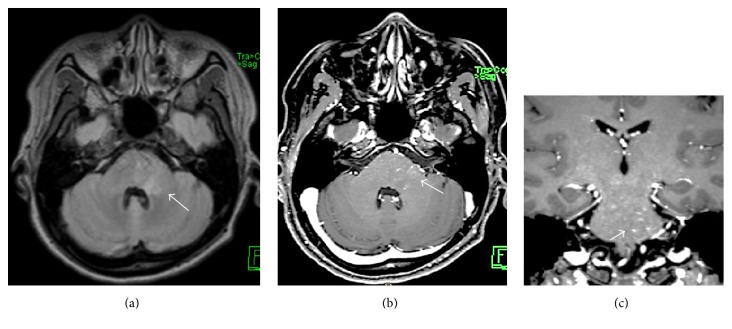
(a) Axial T2/FLAIR and (b) axial and sagittal (c) T1 weighted MRI after IV gadolinium based contrast medium injection, showing ill-defined faint bright intensity in the posterior pons and left brachium pontis (arrow in (a)) and characteristic punctate and linear enhancement (arrow in (b) and (c)).

**Figure 2:**
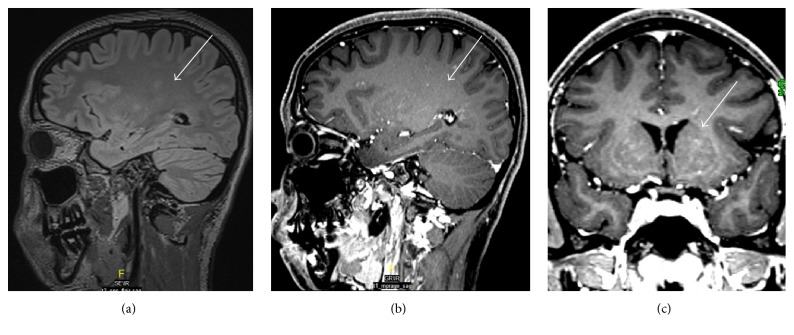
(a) Sagittal T2/FLAIR, (b) sagittal, and (c) coronal T1 weighted MRI after IV gadolinium based contrast medium injection, of the brain, showing white matter ill-defined faint bright intensity (arrows in (a)) and characteristic punctate and linear enhancement in white matter and basal ganglia (arrows in (b) and (c)), respectively.

**Figure 3:**
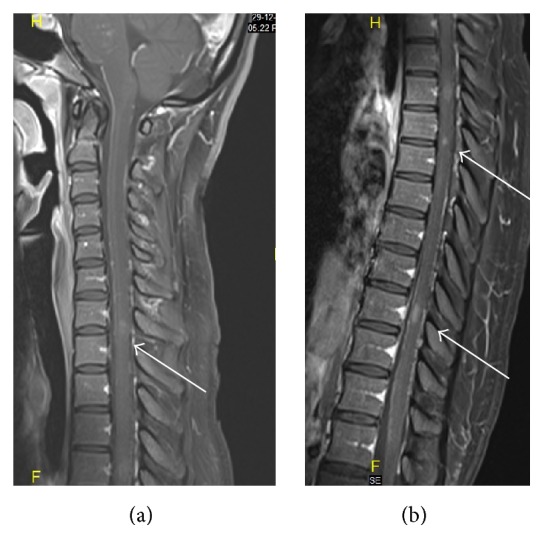
Sagittal T1 weighted MRI after IV gadolinium based contrast medium injection, of the spinal cord, showing multifocal punctate and linear enhancement at cervical and thoracolumbar levels (arrows in (a) and (b)), respectively.

MRI of the brain and spinal cord showed evidence of multiple foci of faint bright signal intensity involving the posterior pons and left brachium pontis (Figure 1), with superior extension extending to cerebral subcortical and deep white matter, bilateral thalami, and basal ganglia (Figure 2), and inferior extension along the spinal cord at multiple levels (Figure 3), with small punctate enhancement that aligned along fine curvilinear enhancing structures; given the typical features, the possibility of CLLIPPERS was raised by the radiologist.

Cerebrospinal fluid study (CSF) showed lymphocytic pleocytosis (WBC: 12, lymphocyte 98%) with normal protein and glucose, and viral serology for HSV1, HSV2, CMV, EBV, VZV, mumps, adenovirus, enterovirus, and parechovirus were negative.

CSF Gram stain and culture were negative. CSF TB PCR and VDRL were nonreactive. Complete blood count and peripheral smear were normal; HIV and Quantiferon TB were also negative. CSF oligoclonal band was negative, in addition to normal visual evoked potential; both made multiple sclerosis unlikely. Antinuclear antibodies were found to be positive (1 : 320, nucleolar in pattern) but anti-double stranded antibodies and anti-cardiolipin antibodies were negative; accordingly the probability of having autoimmune disease without any suggestive symptoms was rather low.

Our patient showed some spontaneous improvement; however, following the institution of steroids with a 60 mg of prednisolone she responded dramatically and started to walk with improvement of balance, nystagmus resolved, and coordination improved significantly. After 2 weeks of prednisolone as outpatient, she returned back to normal functioning, free of any symptom, with completely normal neurological exam.

## 3. Discussion

This patient's clinical and radiological features strongly suggest CLIPPERS syndrome, as described by Simon et al. core features [3]. Our patient shared several clinical and radiological characteristics with the previously described patients.

 Firstly, the clinical symptoms related directly to brainstem involvement, particularly the subacute gait ataxia (seen in all of Pittock et al.'s patients) [1], and nystagmus seen in one patient.

 Secondly, the MRI findings were of multiple, small, patchy T2 weighted hyperintensities, mainly involving the posterior pons and left brachium pontis. In addition to subcortical deep white matter and bilateral thalami, some of them were along enhancing curvilinear structures, likely medullary vessels, suggesting perivascular involvement.

 Thirdly, we needed to exclude various inflammatory, infectious, and paraneoplastic disorders, as well as excluding vasculitis, although our patient had positive ANA, other autoimmune workups were negative, and there were no clinical features suggestive of any vasculitis or any other autoimmune disease; actually there was elevation of autoimmune antibodies in some cases (like in one of the cases previously encountered) [4].

Our patient fulfilled all of these “criteria,” making the diagnosis of CLIPPERS highly probable and justifying the avoidance of brain biopsy. Such a noninvasive approach in “typical cases” was already suggested by Pittock et al., who treated 50% of patients without biopsy.

Unexpectedly patient was found to have positive IgG and IgM antibodies against toxoplasma but actually it is well known that toxoplasmosis is asymptomatic in most of the cases; moreover IgM can be positive up to many years after the initial infection, and also patient's symptoms, radiological picture, and negative HIV make toxoplasmosis unlikely.

 Finally, although our patient showed some spontaneous improvement without any treatment, the addition of glucocorticosteroid accelerated the improvement and patient was found completely asymptomatic in the follow-up visit after two weeks.

## 4. Conclusion

CLIPPERS describes a newly underestimated, misdiagnosed entity affecting the CNS, not clearly defined, and remained a debated not finally clarified issue, and as per the British Society for Immunology, whether it represents an independent, actual new disorder or a syndrome that includes aetiologically heterogeneous diseases, clinicians and radiologists should be aware of this condition and its differential diagnoses, given that CLIPPERS constitutes a treatable condition and that patients may benefit from an early introduction of GCS immunosuppression.
